# Effects of tiletamine-zolazepam vs. propofol on peri-induction intraocular pressure in dogs: A randomized, masked crossover study

**DOI:** 10.3389/fvets.2023.1061755

**Published:** 2023-03-06

**Authors:** Katharine A. McIver, Shannon D. Boveland, Stuart C. Clark-Price, Erik H. Hofmeister

**Affiliations:** Department of Clinical Sciences, College of Veterinary Medicine, Auburn University, Auburn, AL, United States

**Keywords:** intubation, propofol, tiletamine-zolazepam, ophthalmology, induction, intraocular pressure

## Abstract

**Introduction:**

Anesthesia induction agents have the potential to cause severe ocular side effects, resulting in lasting damage to the eye.

**Objectives:**

The purpose of this study is to determine the effects of tiletamine—zolazepam on IOP compared to propofol when they are used as an induction agent in normal healthy dogs.

**Methods:**

Twenty healthy adult client owned dogs weighing 22.2 ± 7.6 kg were selected for the study. In a randomized order, all dogs received tiletamine-zolazepam 5 mg/kg IV or propofol 8 mg/kg IV titrated to effect without premedication. Washout between each treatment was at least seven days. IOP measurements were obtained at four time points: baseline, post-induction, post-intubation, and after recovery using applanation tonometry. No additional procedures were performed. After normality of the data was determined, a linear mixed model was built with time, eye, treatment and all interactions of those variables as fixed effects and subject as a random effect.

**Results:**

There was no significant difference for age, body weight, drug dose, baseline IOP, and recovery IOP between treatments. Average IOP measurements remained within the normal range of 15-25 mmHg at these time points. However, IOP was significantly less elevated by the tiletamine-zolazepam treatment vs. propofol at the post-induction (mean difference: −4.7 ± 4.6 [95%CI −6.8 to −2.5]) and the post-intubation (mean difference: −4.4 ± 4.6 [95%CI −6.5 to −2.2]) time points.

**Clinical significance:**

Dogs receiving tiletamine-zolazepam for anesthetic induction had a significantly less elevated IOP at induction and intubation compared to dogs receiving propofol.

## Introduction

Surgical ocular procedures in dogs require a multitude of special considerations when developing an anesthetic protocol. One of the side effects of some anesthetic drugs is elevation of intraocular pressure (IOP) (1). If pressure within the eye increases, it can worsen existing ocular diseases, such as descemetocele and glaucoma (1). There is the potential for even a mild increase in IOP during the peri-anesthetic period to have ocular side effects, causing lasting damage to the eye and detrimental effects on vision quality. Notably, deep corneal lesions may progress to perforation with increases in IOP (2). Corneal perforations with iridial prolapse have a poorer prognosis regarding vision preservation (3). Furthermore, increases in IOP may further damage the retina and optic nerve in patients with glaucoma (4). It is imperative to minimize the possibility of an elevated IOP in patients with these ocular diseases.

In addition to anesthetic drugs, there are a multitude of factors that influence IOP. Both intraocular and extraocular components contribute to IOP, such as aqueous humor volume, vitreous humor volume, choroidal blood volume, scleral elasticity, extraocular muscle tone, and blood pressure (5). Intraocular pressure is maintained through the balance of production and drainage of aqueous humor within the anterior chamber of the eye (4). Aqueous humor is produced by the ciliary body. It then flows through the pupil and drains *via* the iridocorneal angle and returns to circulation through the scleral venous plexus. Any compromise to the architecture of the eye or production of the aqueous humor may create an imbalance, leading to an increase in IOP and potential loss of vision (4).

Anesthetic sedatives and induction agents may increase, decrease, or have no effect on IOP in dogs (1). Ketamine, a dissociative anesthetic agent, has been shown to increase IOP even when paired with a benzodiazepine (3). Tiletamine is a commonly used dissociative induction agent similar to ketamine (6); however, effects on IOP are not well understood. Jang et al. studied the effects of tiletamine-zolazepam on IOP. In this study, it was found that tiletamine-zolazepam did not have a significant effect on IOP (7). However, this study examined supraclinical doses without masking or randomization. It also did not compare tiletamine-zolazepam with another induction agent with well-studied effects on the IOP. Propofol is known to increase IOP in clinically healthy dogs (8). Hofmeister et al. reports a 26% increase in IOP after induction (9). To the authors knowledge, there is no prospective, masked, randomized clinical trial comparing the IOP effects of tiletamine-zolazepam and propofol. The purpose of this study was to compare the effects of tiletamine-zolazepam to propofol on IOP at the time of induction and recovery from anesthesia in ophthalmologically-normal dogs. The hypothesis was that propofol would cause a greater increase in IOP than tiletamine-zolazepam in clinically normal dogs.

## Methods and materials

### Study design

This was a prospective, randomized, masked, cross-over experimental study. A sample size calculation was performed to detect a difference of 2 mmHg in IOP between groups with a standard deviation of 2.6 mmHg, an alpha of 0.05 and a power of 0.8 (10). The difference of 2 mmHg was chosen as even slight changes in IOP may cause severe complications in patients with ocular disease. The sample size calculated was 14 dogs. This value was increased to 20 to account for patient dropout. Twenty dogs (6 males and 14 females, age range: 1–7 years old, average: 3.6 years old, average weight: 22.2 kg) recruited from College of Veterinary Medicine students, staff, and faculty who had elected to participate were enrolled in the study. Informed client consent was obtained, and clients were incentivized with a $100 gift card.

### Inclusion criteria

Inclusion criteria for dogs were age one to seven years, weighing 7–30 kilograms, and free of systemic and ocular disease. Dogs of brachycephalic conformation were excluded. Prior to the study, all dogs had a standard physical examination and a complete ophthalmic examination. Complete routine ophthalmic exams included slit lamp biomicroscopy and indirect ophthalmoscopy, measurement of IOP, a Schirmer tear test, and fluorescein stain with tear break-up time. These tests were performed on awake patients with no sedation or analgesia by a board-certified veterinary ophthalmologist. No patient objected to the measurement of quality and quantity of the tears for examination of cornea health. A small volume blood sample (3 mL) was acquired directly from the cephalic vein and packed cell volume (PCV), total solids (TS), blood glucose (BG), and blood lactate (BL) were determined. Dogs who had normal findings on all exams and were deemed healthy and free of ocular disease were enrolled in the study. Any dog with abnormalities noted on these tests were excluded.

### Treatments administered

All 20 dogs received both treatments in a randomized crossover study design. Dogs were randomly assigned to receive either tiletamine-zolazepam or propofol first as an anesthetic induction agent. All experiments were performed in the same procedure room at the same time of day. A minimum of 7 days between treatments was used to ensure an adequate washout period. Food was withheld 8 h prior to treatment. Water was available until the scheduled procedure time. Anesthesia was supervised by a licensed veterinarian. Hair on the dorsal aspect of the foreleg was clipped and aseptic preparation of the skin was performed. An appropriately sized intravenous (IV) catheter (22-18 G) was placed in a cephalic vein and secured in place with medical tape. Dogs were induced with an initial dose of 5 mg/kg of tiletamine-zolazepam (Telazol, Zoetis) IV to effect or 8 mg/kg of propofol (PropoFlo 28, Zoetis) administered IV to effect. Dogs were administered 25% of the total volume of either drug every 10 s until loss of jaw tone was noted by the masked licensed veterinary examiner. The dogs were then intubated under direct laryngoscopy with an endotracheal tube size determined by tracheal palpation. One hundred percent oxygen was administered *via* endotracheal tube during anesthesia for both treatments. Heart rate and respiratory rate were monitored *via* auscultation with a stethoscope. Once the dogs recovered their ability to swallow, they were extubated and allowed to fully recover from anesthesia while being monitored. Criteria for full recovery included normothermia (>37.2°C), appropriate mentation, and ability to stand and ambulate normally. The individual judging induction and recovery and performing intubation was masked to treatment allocation. The protocol was approved by the Institutional Animal Care and Use Committee of the university.

### Outcomes measured

The IOP measurements were obtained immediately prior to induction, after induction, after intubation, and upon full recovery The baseline, induction, and intubation time points were separated by five-minute intervals. Time was also recorded from intubation to extubation in five-minute intervals. The IOP was measured using an applanation tonometer (Tono-Pen Vet, Reichert Inc.) which was factory calibrated prior to use in this study. The device was internally calibrated by an ophthalmologist at the start of each research episode. A drop of 0.5% proparacaine hydrochloride ophthalmic solution was applied to the eye to prevent discomfort prior to each IOP measurement. Measurements were taken by the same masked examiner. The IOP values recorded were an average of three readings. Only values within 5% error were recorded. Measurements were taken with the dog in sternal recumbency with their heads in a natural, upright position at the same height with shoulder joint at all time points. Measurements were obtained from the right eye followed by the left eye in all patients.

Statistical analysis was performed using SPSS (IBM). Normality of the data was determined using the Shapiro-Wilk test and visual examination of Q-Q plots. A repeated measures linear mixed model was built with time (baseline, before induction, after intubated, final), eye (right or left), treatment (tiletamine/zolazepam or propofol) and all interactions of those variables as fixed effects and subject as a random effect. Main effects were compared with the least significant difference (LSD) test. A *P* < 0.05 was considered statistically significant.

## Results

**Figure 1:**
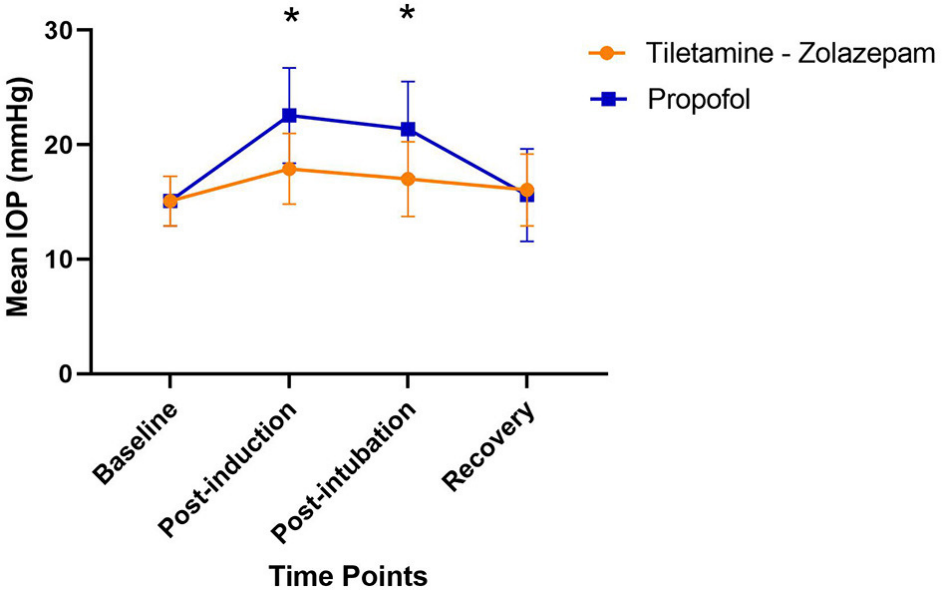
Changes in mean IOP across the induction procedure when treated with tiletamine- zolazepam vs. propofol; ^*^significant difference between groups (*p* < 0.05). Tiletamine- Zolazepam: no significant difference over time; Propofol: post induction mean difference: 7.5 ± 4.2 1 [95%CI −4.4 to −9.5], Post intubation mean difference: 6.3 ± 4.9 [95%CI −3.7 to −7.8]; Tiletamine-zolazepam vs. Propofol: Post-induction mean difference: −4.7 ± 4.6 [95%CI −6.8 to −2.5], Post-intubation mean difference: −4.4 ± 4.6 [95%CI −6.5 to −2.2].

No adverse reactions were noted during IOP measurements or anesthesia. The average time measured between induction and extubation for tiletamine-zolazepam and propofol were 14.25 and 9 min respectively. All patients were noted to have a smooth recovery. There was no significant difference between the right and left eye measurements at any time point. There was no significant difference between age, body weight, drug dose, baseline IOP, and recovery IOP between the groups (Table 1). There was a significant difference in IOP between the two treatment groups at the post-induction and post-intubation time points (*p* < 0.0001) (Table 2). Furthermore, there was a significant difference in IOP in the tiletamine-zolazepam treatment group from baseline to post-induction (mean difference: 2.6 ± 0.6 [95%CI 1.4 to 3.7]) and post-induction to recovery (mean difference: 1.9 ± 0.5 [95%CI 0.8 to 3.0]). However, in the propofol treatment group, there was a significant increase of greater magnitude in IOP from baseline to post-induction (mean difference: 7.5 ± 4.2 [95%CI 10.1–4.9]) and baseline to post-intubation (mean difference: 6.3 ± 4.9 [95%CI 9.4–3.2]). In the propofol group, there was also a significant decrease in IOP from post-induction to recovery (mean difference: −7.0 ± 4.1 [95%CI −4.4 to −9.5]) and post-intubation to recovery (mean difference: −5.8 ± 3.3 [95%CI −3.7 to −7.8]). IOP was significantly less elevated during the tiletamine-zolazepam treatment relative to the propofol at the post-induction (mean difference: −4.7 ± 4.6 [95%CI −6.8 to −2.5]) and the post-intubation time points (mean difference: −4.4 ± 4.6 [95%CI −6.5 to −2.2]) (Figure 1).

**Table 1:** Baseline IOP (mmHg), induction IOP (mmHg), intubation IOP (mmHg), recovery IOP (mmHg), and induction agent dosages (mg/kg).

**Patient number**	**Propofol Dosage (mg/kg)**	**Baseline IOP (mmHg)**	**Induction IOP (mmHg)**	**Intubation IOP (mmHg)**	**Recovery IOP (mmHg)**
1	8.0	15.5	17	15	17
2	6.9	17.0	19.5	17	12
3	6.2	15.0	26.5	24.5	16.5
4	6.8	14.0	25.5	21	14
5	4.3	12.0	17	18.5	12
6	5.9	20.0	24.5	24	17
7	5.5	18.0	25.5	18	13
8	6.8	18.0	30	25	15
9	6.9	13.5	25.5	28	20
10	6.8	15.0	21	18.5	17
11	7.8	15.5	30	29	22.5
12	6.3	12.5	19	20.5	14
13	8.0	15.0	14.5	15	12.5
14	5.9	14.0	20.5	19.5	11.5
15	5.6	12.0	23.5	23.5	15.5
16	8.0	13.5	25.5	27	28
17	5.1	18.0	20.5	17.5	13.5
18	8.0	14.5	23.5	20	13.5
19	7.1	14.0	21	24.5	13.5
20	8.0	14.5	21	21	14
**Patient number**	**Tiletamine-zolazepam Dosage (mg/kg)**	**Baseline IOP (mmHg)**	**Induction IOP (mmHg)**	**Intubation IOP (mmHg)**	**Recovery IOP (mmHg)**
1	5.0	16	14.5	11.5	11.5
2	3.8	17.5	17.5	18.5	16.5
3	2.8	14.5	17	22	17
4	5.0	14.5	16	19.5	16.5
5	5.0	12.5	19.5	18.5	22
6	5.0	20.5	21.5	19	20.5
7	4.1	18.5	21	16	17
8	5.0	18.5	21.5	22	18.5
9	5.0	14	23	21.5	20
10	5.0	14.5	15.5	14.5	11.5
11	5.0	15	18	12.5	13
12	5.0	13	14	13	17
13	5.0	15.5	19.5	16	14.5
14	5.0	13.5	20.5	19	18
15	5.0	12.5	19	17	15
16	5.0	14	17	18	18
17	5.0	17.5	19.5	19	17.5
18	4.1	15	10.5	14.5	11.5
19	4.0	14.5	14.5	12	12
20	4.3	15	18.5	16	13.5

**Table 2:** Number of eyes that had an increase (and the extent to which it increased), decrease or no change following induction with tiletamine-zolazepam (*n* = 20 dogs), or propofol (*n* = 20 dogs) and mean ± SD of the delta in intraocular pressure between baseline and immediately following anesthetic induction.

**Parameter**	**Tiletamine-zolazepam**	**Propofol**
IOP increase	18	19
IOP decrease	2	1
IOP no change	0	0
**IOP increase in:**		
1–2 mmHg	9	3
3–4 mmHg	4	1
5–6 mmHg	2	5
7–8 mmHg	2	2
9–10 mmHg	1	1
>10 mmHg	0	7
Delta IOP (mmHg)	2.8 ± 3.1	7.5 ± 4.2^*^

* Significantly different from the tiletamine-zolazepam group (p < 0.05).

## Discussion

The current study investigated changes in IOP in healthy dogs following induction with tiletamine-zolazepam or propofol. Treatment with tiletamine-zolazepam resulted in a significantly less elevated IOP when compared to propofol at both induction and intubation. These results are in accordance with the findings of previous studies.

Propofol and its effects on IOP have been previously researched. Hofmeister et al. investigated the effects of propofol vs. thiopental on IOP in normal canines (11). Their findings concluded that propofol increased IOP by 26% prior to intubation (11). Furthermore, it remained increased by 24% after intubation. They concluded that propofol should be avoided, when possible, in patients where an increase in IOP would be harmful (11). In a subsequent study, Hofmeister et al. compared graded doses of propofol and their effects on IOP (9). The increase in IOP was consistent with the previous study but did not appear to be dose dependent (9).

Jang et al. compared changes in IOP during induction across different dosages of IV tiletamine-zolazepam ranging from 5 mg/kg to 20 mg/kg and reported no significant difference between baseline IOP and post-treatment IOP (7). This study had a small sample size, lacked masking and randomization, and tested supraclinical doses. In the current study, clinically applicable dosages of tiletamine-zolazepam were used along with masking and randomization. The findings of this study showed that tiletamine-zolazepam does elevate IOP a significant amount but to a lesser magnitude than propofol.

Smith et al. examined the effects of ketamine-diazepam and propofol on IOP in combination with premedication of dexmedetomidine and hydromorphone over 40 min under general anesthesia. Findings showed that both treatments increased IOP after induction and abolished the predicted decreased IOP caused by the premedication (12). Since ketamine-diazepam and tiletamine-zolazepam are analogs and share a close chemical relationship (6), it is of note that IOP was less elevated from baseline after induction with tiletamine-zolazepam. It is important to note that the benzodiazepine used with the induction agents was different between the present investigation and the work of Smith et al. (zolazepam vs. diazepam). Hahnenberger reports administration of zolazepam lowered IOP by ~10% in cats (13). This lends support to the notion that the type of benzodiazepine used with the primary induction agent may influence the strength of effect observed with dissociative agents on IOP. Future studies are warranted to further understand how the type of benzodiazepine paired with the dissociative may contribute to changes in IOP.

In the present study, IOP returned to baseline under both treatment conditions after recovery from the induction agents. This is likely due to the rapid redistribution characteristics of both agents. Tiletamine is chemically related to ketamine as both agents are N-methyl-D-aspartate receptor antagonists used as a dissociative anesthesia (6). Zolazepam, a non-phenothiazine diazepinone similar to diazepam and midazolam, is used in conjunction with tiletamine to mitigate the muscle rigidity and sympathetic stimulation noted (14). The average half-life of tiletamine-zolazepam is 40 min (6). Propofol is a lipophilic hypnotic drug that is known for its rapid smooth induction and recovery (15). It has a biphasic clearance, with an initial half-life of ~30 min (15). Due to its lipophilic properties, it rapidly crosses the blood brain barrier and redistributes rapidly, mitigating its clinical effects.

As previously mentioned, there are many factors that contribute to IOP. Not all factors, most notably PaCO_2_ and blood pressure, were able to be measured in this study. Elevation in PaCO_2_ has been shown to increase IOP (16). The current study did not include general anesthesia with further time points as previous studies have shown that inhalant anesthetics have minimal effects on IOP (17). Furthermore, the addition of premedications was not included in the study design. Webb et al. examined the IOP effects of propofol four populations: premedicated heathy dogs, premedicated dogs with glaucoma, non-premedicated healthy dogs, and non-premedicated dogs with glaucoma (10). Findings included an increase in IOP in premedicated and non-premedicated healthy dogs, though the response was blunted in the premedicated group. Even though these are important elements of IOP, the focus of this study was to directly compare tiletamine-zolazepam and propofol during the induction and intubation phase of anesthesia. By focusing on this short period of time and one variable, the goal was to generate data that will aid practitioners in induction drug selection during the peri-anesthetic phase. Further studies comparing the IOP effects of tiletamine-zolazepam and propofol in premedicated patients is warranted.

Veterinary practitioners have a variety of anesthetic induction drugs to utilize in their practice. It is important to consider the possible side effects of these induction agents when creating a tailored anesthetic protocol. Tiletamine-zolazepam and propofol are commonly used induction agents in veterinary medicine. They both provide a rapid smooth induction for general anesthesia and are well tolerated in healthy animals. Special consideration should be taken in patients with ocular disease. Specifically, IOP effects of these drugs should be noted by veterinarians during use in their daily practice.

In conclusion, induction with tiletamine-zolazepam lead to significantly less elevated IOP compared to induction with propofol in healthy dogs. Given the potential danger posed to animals at risk of ocular damage from increased IOP during a surgical procedure, results from this study have direct clinical implications for considerations on the appropriate induction agent to be used. Further studies are necessary to fully understand the effects of induction agents on IOP as well as how the type of benzodiazepine used with the dissociative may influence effects on IOP, particularly in populations at higher risk of ocular damage.

## Data availability statement

The original contributions presented in the study are included in the article/supplementary material, further inquiries can be directed to the corresponding author.

## Ethics statement

The animal study was reviewed and approved by Auburn University IACUC Committee. Written informed consent was obtained from the owners for the participation of their animals in this study.

## Author contributions

KM and EH conceptualized and designed the experiment. KM, EH, and SB performed the experiment. KM analyzed the data and drafted the manuscript. KM, EH, SB, and SC-P participated in manuscript revision. All authors approved the final version of the manuscript.
